# Characterization of astrocytes throughout life in wildtype and APP/PS1 mice after early-life stress exposure

**DOI:** 10.1186/s12974-020-01762-z

**Published:** 2020-03-20

**Authors:** Maralinde R. Abbink, Janssen M. Kotah, Lianne Hoeijmakers, Aline Mak, Genevieve Yvon-Durocher, Bram van der Gaag, Paul J. Lucassen, Aniko Korosi

**Affiliations:** Brain Plasticity Group, Center for Neuroscience, Swammerdam Institute for Life Sciences, University of Amsterdam, Science Park 904, 1098 XH Amsterdam, The Netherlands

**Keywords:** GFAP, Early stress, Aβ pathology, APP/PS1, Astrocytes, Glia

## Abstract

**Background:**

Early-life stress (ES) is an emerging risk factor for later life development of Alzheimer’s disease (AD). We have previously shown that ES modulates amyloid-beta pathology and the microglial response to it in the APPswe/PS1dE9 mouse model. Because astrocytes are key players in the pathogenesis of AD, we studied here if and how ES affects astrocytes in wildtype (WT) and APP/PS1 mice and how these relate to the previously reported amyloid pathology and microglial profile.

**Methods:**

We induced ES by limiting nesting and bedding material from postnatal days (P) 2–9. We studied in WT mice (at P9, P30, and 6 months) and in APP/PS1 mice (at 4 and 10 months) (i) GFAP coverage, cell density, and complexity in hippocampus (HPC) and entorhinal cortex (EC); (ii) hippocampal gene expression of astrocyte markers; and (iii) the relationship between astrocyte, microglia, and amyloid markers.

**Results:**

In WT mice, ES increased GFAP coverage in HPC subregions at P9 and decreased it at 10 months. APP/PS1 mice at 10 months exhibited both individual cell as well as clustered GFAP signals. APP/PS1 mice when compared to WT exhibited reduced total GFAP coverage in HPC, which is increased in the EC, while coverage of the clustered GFAP signal in the HPC was increased and accompanied by increased expression of several astrocytic genes. While measured astrocytic parameters in APP/PS1 mice appear not be further modulated by ES, analyzing these in the context of ES-induced alterations to amyloid pathology and microglial shows alterations at both 4 and 10 months of age.

**Conclusions:**

Our data suggest that ES leads to alterations to the astrocytic response to amyloid-β pathology.

## Background

Early-life stress (ES) has been associated with cognitive deficits in adulthood [1–4], and there is emerging evidence that ES also increases the risk for developing Alzheimer’s disease (AD) [5, 6]. For example, among children who have experienced physical neglect, mild cognitive impairment, considered a prodromal phase of AD, is more frequently observed [7]. Moreover, parental loss and stress during childhood have been associated with an increased risk of developing AD [8–10]. The use of pre-clinical ES models has provided important insights into some of the mechanisms that might mediate ES-induced increases in AD risk. For example, ES in different rodent models modulates the aggregation of amyloid beta (Aβ), one of the neuropathological hallmarks of AD [11]. ES further triggers an earlier onset of Aβ plaques [12], and increases Aβ plaque load [12–14], which is accompanied by exacerbated cognitive impairment and shorter survival in AD mouse models [12, 13, 15, 16], but see [17], where ES did not further exacerbate cognitive impairments, indicative of floor effects, which highlights the importance of the specific tasks, timing, and protocols performed.

Astrocytes are one of the most studied cell types in the context of AD. In fact, in response to Aβ pathology, astrocytes acquire a reactive phenotype characterized by upregulation of glial fibrillary acidic protein (GFAP) (i.e., astrogliosis) [18, 19]. Such alterations in astrocyte reactivity have been consistently shown in both AD patients [18, 20–22] and rodent models of AD [23, 24]. Astrocytes are vital for synaptogenesis [25–27], which is highly disrupted by AD pathology [28–30] (for review see [31]), and is strongly correlated with cognitive decline [32].

Interestingly, there is emerging evidence that astrocytes are also implicated in effects of ES (reviewed in [33]). ES leads to immediate and permanent changes in astrocyte number and morphology [34–38] that may contribute to cognitive deficits. Furthermore, we have shown that ES leads to persistent alterations in microglia up to the age of 10 months. Microglia are essential in the immune response to Aβ [39] and exhibit an aggravated inflammatory response to Aβ pathology in AD mice previously exposed to ES [14]. However, astrocytes are also crucial in governing inflammatory responses, and astrocytes and microglia closely interact during AD-related neuroinflammation [40–43]. For example, both microglia and astrocytes cluster around accumulating Aβ in clinical and pre-clinical brains [11]. Furthermore, Aβ pathology in AD mice triggers cytokine release from both cell types [44], as well as a pro-inflammatory transcriptome, the latter effect being more pronounced in astrocytes [45].

We therefore set out to study the involvement of astrocytes in the ES-induced modulation of later AD pathology. We hypothesize that ES persistently affects astrocytes and may thereby contribute to the aggravation of AD pathology following ES exposure. We present here (i) effects of ES on astrocytes across different ages (P9, P30, and 6 months), (ii) effects of ES on the astrocytic response to Aβ accumulation during early (4 months) and advanced (10 months) pathological stages in the hippocampus (HPC) and entorhinal cortex (EC), and (iii) how these measures relate to the Aβ pathology and microglial markers previously reported in the same cohort [14].

## Methods

### Animals and breeding

C57Bl/6 J and bigenic APPswe/PS1dE9 hemizygous animals on a C57Bl/6 J background were used. APP/PS1 mice express mutated versions of the APP and PS1 proteins under the mouse prion promoters, containing a chimeric mouse/human amyloid precursor protein with the Swedish mutation (K595N/M596L) and the presenilin-1 protein with a deletion on exon 9 [46, 47]. Experimental animals were bred in house as described previously [48]. In total, three cohorts of male WT C57Bl/6 J mice of different ages (P9, P30, and 6 months) and two cohorts of male WT/APP/PS1 mice of different ages (4 months and 10 months) were used for experiments. All mice were kept under standard housing conditions (temperature 20–22 °C, 40–60% humidity level, chow/water ad libitum, 12/12 h light/dark schedule). Experiments were approved by the Animal Experiment Committee of the University of Amsterdam and performed in accordance to European Union (EU) directive 2010/63/EU.

### Early-life stress paradigm

The ES paradigm consisted of limiting nesting and bedding material from P2 to P9 as described previously [48]. Briefly, at P2, litters were randomly assigned to control (CTL) or ES condition. CTL litters received standard nesting and bedding material. ES litters were placed on a fine-gauge stainless-steel mesh positioned 1 cm above the cage floor with half the amount of nesting material. At P9, pups were moved to standard cages or sacrificed (P9 cohort). This ES model has been shown to lead to decreased body weight gain, increased adrenal weights, and decreased dentate gyrus volume in pups at P9 [48]. We assessed all of these also in our current cohort to confirm the effectiveness of the ES exposure. Dentate gyrus volume was measured using the Cavalieri principle, by multiplying the total area measured in ImageJ with the thickness of the slice, as well as the number of parallel series created per brain.

### Tissue preparation

Mice were sacrificed via transcardial perfusion (P9, P30, 4, 6, and 10 months) or rapid decapitation (P9, 4, and 10 months), and tissue was harvested for either immunohistochemical or gene expression analyses, respectively. To collect brain material for immunohistochemical purposes, mice (P9: WT-CTL *n* = 7, WT-ES *n* = 6; P30: WT-CTL *n* = 4, WT-ES *n* = 6; 4 months: WT-CTL *n* = 10, WT-ES *n* = 11, APP/PS1-CTL *n* = 7, APP/PS1-ES *n* = 7; 6 months: WT-CTL *n* = 4, WT-ES *n* = 8; 10 months: WT-CTL *n* = 7, WT-ES *n* = 8, APP/PS1-CTL *n* = 5, APP/PS1-ES *n* = 4) were anesthetized via an intraperitoneal injection of pentobarbital (120 mg/kg Euthasol®) and transcardially perfused with 0.9% saline followed by 4% paraformaldehyde in phosphate buffer (PB 0.1 M, pH 7.4). The brains were harvested as previously described [14], sliced in 40 μm thick coronal sections (4 parallel series for P9, 6 parallel series for adult) and stored in antifreeze solution (30% Ethylene glycol, 20% Glycerol, 50% 0.05 M PBS) at − 20 °C until further processing. To collect the brain material for gene expression analyses, mice (P9: WT CTL *n* = 8, WT ES *n* = 5; 4 months: WT-CTL *n* = 7, WT-ES *n* = 6, APP/PS1-CTL *n* = 5, APP/PS1-ES *n* = 4; 10 months: WT-CTL *n* = 6, WT-ES *n* = 11, APP/PS1-CTL *n* = 6, APP/PS1-ES *n* = 6) were sacrificed via rapid decapitation and hippocampi were quickly dissected and snap-frozen on dry ice. RNA was extracted from hippocampi using the TRIzol method (TRIzol, Invitrogen). Reverse transcription of RNA to cDNA was performed using SuperScript® III Reverse Transcriptase (Invitrogen), and cDNA samples were stored at − 20 °C until further processing.

### Immunohistochemistry

Free-floating brain sections were incubated with primary (polyclonal rabbit anti-GFAP, 1:10000, Dako) and secondary antibody (goat anti-rabbit, 1:200, Vector biotinylated), followed by 90 min incubation with avidin-biotin complex (ABC elite kit, 1:800, Vectastain, Brunschwig Chemie). Subsequently, for chromogen development, sections were incubated for 20 min with 0.5 mg/mL 3,3′-diaminobenzidine (DAB) with 0.01% H2O2 in 0.05 M TB. After DAB staining, sections were mounted on pre-coated glass slides (Superfrost Plus slides, Menzel) followed by coverslipping. Volume estimations of the P9 DG were obtained by measuring DG tracings of 6 bilateral sections to estimate the total surface area in square micrometers, which was multiplied by the number of series (4) and section thickness (40 μm).

### RT-qPCR

Relative gene expression was assessed via PCR amplification of cDNA using the Hot FirePol Eva-green qPCR supermix (Solis Biodyne) and measured using the 7500 Real-time PCR system (Applied Biosystems). Primer sequences are listed in Table 1 and obtained 90–110% efficiency. Relative gene expression was calculated using the ΔΔ*C*_t_ method in qBASE (Biogazelle) after normalization to at least two stable (*M* < 0.5, CV < 0.25) reference genes (RPL13A, RPL0, SDHA) not altered by experimental conditions [49, 50]. These experiments were done in triplicate, with *C*_T_ values within 0.5 of each other being averaged for analyses.

**Table 1:** Primer sequences for RT-qPCR

Target gene	Forward primer (5′-3′)	Reverse primer (5′-3′)
Housekeeping genes		
Rpl13a	CCCTCCACCCTATGACAAGA	TCGCCTGTTTCCGTAACCTC
Rpl0	GCTTCATTGTGGGAGCAGACA	CATGGTGTTCTTGCCCATCAG
Sdha	GTTGCTGTGTGGCTGACTG	GCACAGTGCAATGACACCAC
Astrocyte genes		
Aldh1l1	GCAGGTACTTCTGGGTTGCT	GGAAGGCACCCAAGGTCAAA
Apq4	ATCAGCATCGCTAAGTCCGT	ATCCTCCAACCACACTGGGA
Fasn	GACTCGGCTACTGACACGAC	CGAGTTGAGCTGGGTTAGGG
Gfap	ACAGAGGAGTGGTATCGGTCT	GGACTCCAGATCGCAGGTCA
Glast	GATTTGCCCTCCGACCGTAT	CGCCATTCCTGTGACGAGAC
Glt1	CATGTCCACGACCATCATTGC	AGGCTAGACACCTCGTCGTT
GluS	CCACCGCTCTGAACACCTT	ACTCTTCCACACACTTGGGC
Vimentin	ACTGCACGATGAAGAGATCCAG	CACGCTTTCATACTGCTGGC

### GFAP quantification

GFAP+ astrocyte coverage (global and clustered GFAP), cell density, and individual cell complexity were analyzed in immunostained sections. For coverage and cell density analyses, sections were imaged with a 10x objective on a Nikon Eclipse light microscope. To ensure representative analyses of the whole hippocampus, we took rostro-caudal sections located between the bregma levels − 1.34 to − 3.80. We used 6 slices in the P9 samples and 7–8 slices in the P30, P180, and 10-month samples. In analyzing the EC in 4-month and 10-month samples, we took 2 sections between the bregma − 3.64 to − 4.16. Whole HPC and hippocampal subregions dentate gyrus (DG), stratum lacunosum-moleculare (SLM), and cornu ammonis (CA), (CA1, CA2, CA3 separately for P9 cohort) and the EC (4 months and 10 months WT/APP/PS1) were traced using ImageJ software. After tracing, images were converted to 8-bit black-and-white images in ImageJ. A fixed threshold was determined for each cohort to determine the percentage of immunoreactive stained area (coverage). The total thresholded signals and areas of interest across different bregma points analyzed were averaged, producing a single coverage datapoint per animal. In order to measure clustered GFAP, thresholded images were processed in ImageJ using the Analyze Particles function. GFAP signal with a surface area below 1400 μm^2^ was filtered out to obtain clusters of GFAP, and the coverage analysis was repeated on the processed images. Data are presented as percentage coverage.

Astrocyte density was obtained by performing manual cell counts in 4 selected frames of 6744 μm^2^ in the hippocampal subregions of interest (hilus, molecular layer (ML) of the DG, SLM, and CA1). Data are presented as number of GFAP+ cells per surface area. For cell complexity measurements, Z-stack images were obtained with a 40x objective. For each animal, 6 cells in 4 coronal sections (bregma levels − 1.70 until − 2.80) were used for cell complexity measurements, resulting in a total of 24 analyzed cells per animal. The outline of individual cells was traced to obtain a 2D cell surface. Sholl analysis was performed on traced cells using ImageJ [51, 52]. Virtual concentric circles where drawn with a 2 μm radius interval from the soma for a total distance of 74 μm, and the number of intersections was counted. The number of primary processes was counted manually.

Immunohistochemical stainings of GFAP in 4 and 10-month WT/APP/PS1 mice were done on parallel series from the same brains used in our previous study [14], where we characterized amyloid pathology and the microglial markers Iba1 and CD68. This allowed us to normalize the GFAP data to measures of amyloid. In addition to further explore interactions between astrocytes, microglia, and amyloid pathology in the HPC of WT and APP/PS1 mice, we created correlation matrices with the data obtained from different immunostainings (GFAP, CD68, Iba1, 6E10) in 4-month and 10-month-old mice. These descriptive correlations aimed to explore the relation between astrocytes, microglia, and amyloid.

### Statistical analysis

Data were analyzed using SPSS 20.0 (IBM software), Graphpad Prism 5 (Graphpad software), and R 3.5.1 [53]. Data are expressed as mean ± standard error of the mean (SEM) and were considered statistically significant when *p* < 0.05. As multiple mice from the same litter were included in our experiments, the contribution of litter was tested in a mixed model with litter included as a random factor. Litter effects were corrected when present. Data with only condition (CTL/ES) as predictor variable was analyzed with unpaired Student’s *t* test when passing parametric assumptions and Mann-Whitney *U* test otherwise. Data with both condition (CTL/ES) and genotype (WT/APP/PS1) as predictor variables were analyzed using two-way ANOVA. In case of significant interaction effects, post hoc analyses were performed using Tukey’s post hoc test. For data from the Sholl analyses, where multiple cells from one animal were analyzed, the contribution of animal was tested and corrected for as well. Differences between areas under the curve (AUC) and the number of primary processes were analyzed by fitting into a linear model while correcting for nested within animal effects using the *nlme* package in R [54]. Sholl analyses bar graphs and correlation matrices were generated using the ggcorrplot package [55] in R. Pearson correlations were calculated based on complete pairwise cases. Correlation coefficients were tested against critical values on a two-tailed distribution (alpha = 0.05) based on the number of complete cases per comparison. Correlation plots were generated via the ggplot2 package [56] in R.

## Results

### ES increases GFAP expression at P9 in WT mice, which disappears at P30 and 6 months

ES led to physiological signs of stress in the pups, including decreased body weight gain between P2 and P9 (*t* (7) = 4.596, *p* = 0.002), increased adrenal gland weight (*t* (9) = − 3.591, *p* = 0.006), and decreased DG volume at P9 (*t* (11) = 2.368, *p* = 0.037) (data not shown).

**Fig. 1:**
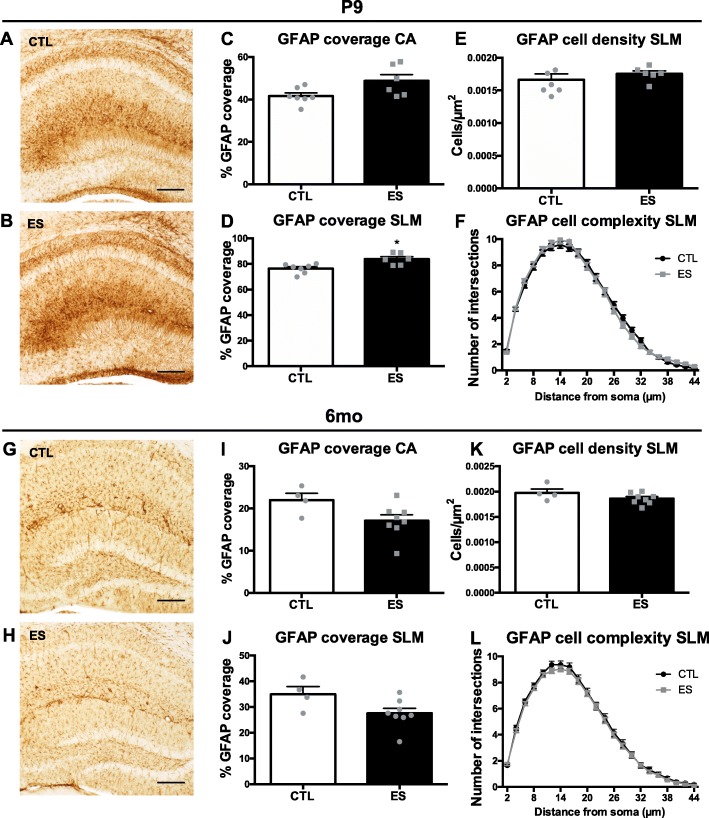
Characterizing ES effects on hippocampal GFAP expression in different developmental stages of WT mice. Representative images of GFAP expression in the HPC of P9 CTL (**a**) and ES (**b**) mice. Insets depict GFAP+ cells at 100x magnification in the SLM. At P9, ES does not affect GFAP coverage in the CA (**c**) but increases GFAP coverage in the SLM (**d**). No differences in GFAP+ cell density (**e**) or GFAP+ cell complexity (**f**) were found in the SLM region. Representative images of GFAP expression in the HPC of 6-month CTL (**g**) and ES (**h**) mice. Insets depict GFAP+ cells at × 100 in the SLM. GFAP coverage is not affected by ES at this age in the CA (**i**) and SLM (**j**). No differences in GFAP+ cell density (**k**) or GFAP+ cell complexity (**l**) were found in the SLM region. Statistical analyses were performed using independent *t* tests for GFAP coverage and cell density and repeated measures ANOVA for cell complexity. ^*^Significant effect of condition *p* < 0.05; scale bars: representative images 250 μm, inset images 12.5 μm

The effect of ES on astrocyte GFAP coverage, GFAP+ cell density, and GFAP+ cell complexity in WT animals was analyzed across different ages. At P9, there was significantly increased GFAP coverage in the SLM, which was unaffected in the other hippocampal subregions (Fig. 1a (CTL) and Fig. 1b (ES); HPC: *t* (7.095) = − 2.029, *p* = 0.082; DG: *t* (11) = − 1.876, *p* = 0.087; CA: *t* (7.372) = − 2.190, *p* = 0.063 Fig. 1c; SLM *t* (11) = − 3.289, *p* = 0.007 Fig. 1d). No differences in GFAP+ cell numbers were found (hilus: *t* (11) = − 1.158, *p* = 0.272; CA1: *t* (11) = − 0.458, *p* = 0.656; SLM: *t* (11) = − 0.869, *p* = 0.403 Fig. 1e), nor could differences in cell complexity be demonstrated (number of intersections AUC: *t* (10) = 0.099, *p* = 0.923 Fig. 1f; number of primary processes: *t* (10) = − 0.6071, *p* = 0.557) in the SLM of P9 mice. Gene expression analyses revealed no changes in mRNA expression of any of the astrocyte markers between CTL and ES at *P*9 (see Table 2).

**Table 2:** Gene expression results. Each row is a tested gene; each column indicates the *p* value for condition, genotype, and interaction effects, and direction of change when *p* < 0.05

	P9 gene expression	4-month gene expression			10-month gene expression		
Gene	ES	ES	APP/PS1	Interaction	ES	APP/PS1	Interaction
Aldh1l1	*p* = 0.118	*p* = 0.220	*p* = 0.083	*p* = 0.353	*p* = 0.141	*p* = 0.001*↓	*p* = 0.955
Aqp4	*p* = 0.812	*p* = 0.822	*p* = 0.830	*p* = 0.345	*p* = 0.243	*p* < 0.001*↑	*p* = 0.593
Fasn	*p* = 0.914	*p* = 0.047*↑	*p* = 0.977	*p* = 0.636	*p* = 0.814	*p* = 0.283	*p* = 0.733
Gfap	*p* = 0.351	*p* = 0.372	*p* = 0.606	*p* = 0.544	*p* = 0.197	p < 0.001*↑	*p* = 0.103
Glast	*p* = 0.816	*p* = 0.846	*p* = 0.370	*p* = 0.378	*p* = 0.453	*p* = 0.149	*p* = 0.359
Glt1	*p* = 0.079	*p* = 0.315	*p* = 0.364	*p* = 0.863	*p* = 0.151	*p* = 0.255	*p* = 0.312
GluS	*p* = 0.686	*p* = 0.268	*p* = 0.932	*p* = 0.992	*p* = 0.989	*p* = 0.054	*p* = 0.977
Vimentin	*p* = 0.913	*p* = 0.114	*p* = 0.708	*p* = 0.618	*p* = 0.468	*p* = 0.024*↑	*p* = 0.975

At P30, no differences in GFAP coverage were found in the whole HPC or hippocampal subregions (HPC: *t* (8) = − 0.995, *p* = 0.349; DG: *t* (8) = − 0.721, *p* = 0.492; CA: *t* (8) = − 1.070, *p* = 0.316).

At 6 months, there were no differences in the HPC and its subregions in GFAP coverage (Fig. 1g (CTL) and Fig. 1h (ES) HPC: *t* (10) = 2.054, *p* = 0.067; DG: *t* (10) = 1.909, *p* = 0.085; CA: *t* (10) = 2.127, *p* = 0.059 Fig. 1i; SLM: *t* (10) = 2.128, *p* = 0.059 Fig. 1j). A decrease in GFAP+ cell density after ES was found in the hilus (*t* (10) = 3.169, *p* = 0.010) but not in other hippocampal subregions (CA1: *t* (10) = − 0.956, *p* = 0.362; SLM: *t* (10) = 1.472, *p* = 0.172 Fig. 1k; DG: *t* (10) = 0.773, *p* = 0.457). Furthermore, no alteration in cell complexity was found at 6 months (number of intersections AUC: *t* (9) = − 0.711, *p* = 0.495 Fig. 1l; primary processes: *t* (9) = − 1.753, *p* = 0.114).

### ES and amyloid pathology do not affect expression of GFAP and astrocyte-related genes at 4 months

**Fig. 2:**
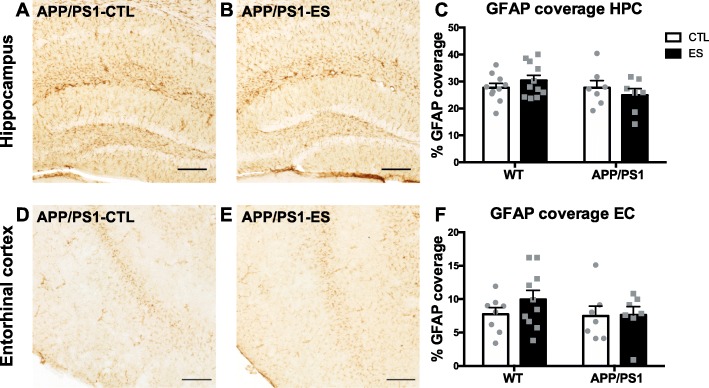
ES does not affect GFAP expression in the HPC or EC of 4-month-old-mice. Representative images of GFAP expression in the HPC of APP/PS1-CTL (**a**) and APP/PS1-ES (**b**) mice and the EC of APP/PS1-CTL (**d**) and APP/PS1-ES (**e**) mice. Both ES and APP/PS1 did not alter GFAP expression in either the HPC (**c**) or EC (**f**) of 4-month-old mice. Statistical analyses were performed using two-way ANOVA. Scale bars 250 μm

In Fig. 2, example images of GFAP coverage of 4-month-old transgenic mice are shown for the HPC: APP/PS1-CTL (Fig. 2a), APP/PS1-ES (Fig. 2b), and the EC: APP/PS1-CTL (Fig. 2d), APP/PS1-ES (Fig. 2e). At 4 months, there was no difference in GFAP coverage induced by either ES or APP1/PS1 overexpression in hippocampal subregions or the EC (HPC: *F*_condition_(1, 31) < 0.000, *p* = 0.986, *F*_genotype_(1, 31) = 1.650, *p* = 0.208, *F*_condition*genotype_(1, 31) = 1.700, *p* = 0.202 Fig. 2c; DG: *F*_condition_(1, 31) = 0.094, *p* = 0.761, *F*_genotype_(1, 31) = 0.967, *p* = 0.333, *F*_condition*genotype_(1, 31) = 1.425, *p* = 0.242; CA: *F*_condition_(1, 31) = 0.059, *p* = 0.810, *F*_genotype_(1, 31) = 1.690, *p* = 0.203, *F*_condition*genotype_(1, 31) = 1.879, *p* = 0.180; EC: *F*_condition_(1, 28) = 0.807, *p* = 0.377, *F*_genotype_(1, 28) = 0.982, *p* = 0.330, *F*_condition*genotype_(1, 28) = 0.604, *p* = 0.444 Fig. 2f).

Gene expression analyses show that ES increased Fasn gene expression levels at 4 months without affecting the other genes that were analyzed (see Table 2).

### The effects of amyloid pathology and ES in the EC and HPC of 10-month-old mice

#### Amyloid pathology increases GFAP expression in the EC at 10 months, which is not further affected by ES

**Fig. 3:**
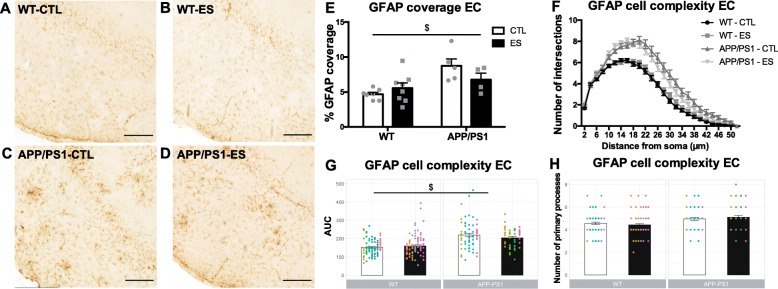
APP/PS1 overexpression increases GFAP coverage and complexity in the EC of 10-month-old mice. Representative images of the EC of 10-month-old WT-CTL (**a**), WT-ES (**b**), APP/PS1-CTL (**c**), and APP/PS1-ES (**d**) mice. GFAP coverage in the EC was increased in APP/PS1 mice (**e**). APP/PS1 increased GFAP+ cell complexity in the EC as shown by increased number of intersections (**f**), area under the curve (AUC) (**g**), and number of primary processes (**h**). Dots in Sholl analysis graphs are color-coded per animal. Statistical analyses were performed using two-way ANOVA. ^$^Significant effect of genotype *p* < 0.05, scale bars 250 μm

In Fig. 3, example images of GFAP coverage of 10-month-old mice are shown for the EC of WT-CTL (Fig. 3a), WT-ES (Fig. 3b), APP/PS1-CTL (Fig. 3c), and APP/PS1-ES (Fig. 3d). Clustering of GFAP is observed in APP/PS1 but not WT mice. In the EC, APP/PS1 causes a global increase in GFAP coverage that is not further affected by ES (EC: *F*_condition_(1, 20) = 0.488, *p* = 0.493, *F*_genotype_(1, 20) = 12.002, *p* = 0.002, *F*_condition*genotype_(1, 20) = 3.447, *p* = 0.078 Fig. 3e). Furthermore, APP/PS1 increased cytoskeletal complexity of GFAP+ cells in the EC, as measured by the area under the curve (*T*_condition_(1, 19) = 0.898, *p* = 0.3805, *T*_genotype_(1, 19) = 5.425, *p* < 0.001, *T*_condition*genotype_(1, 19) = − 1.397, *p* = 0.179 Fig. 3f, g), but did not change the number of primary processes (*T*_condition_(1, 20) = − 0.649, *p* = 0.524, *T*_genotype_(1, 20) = 1.690, *p* = 0.107, *T*_condition*genotype_(1, 20) = 0.857, *p* = 0.401 Fig. 3h).

#### Effects of amyloid pathology and ES on global and clustered GFAP expression and astrocyte-related gene expression in the HPC at 10 months

**Fig. 4:**
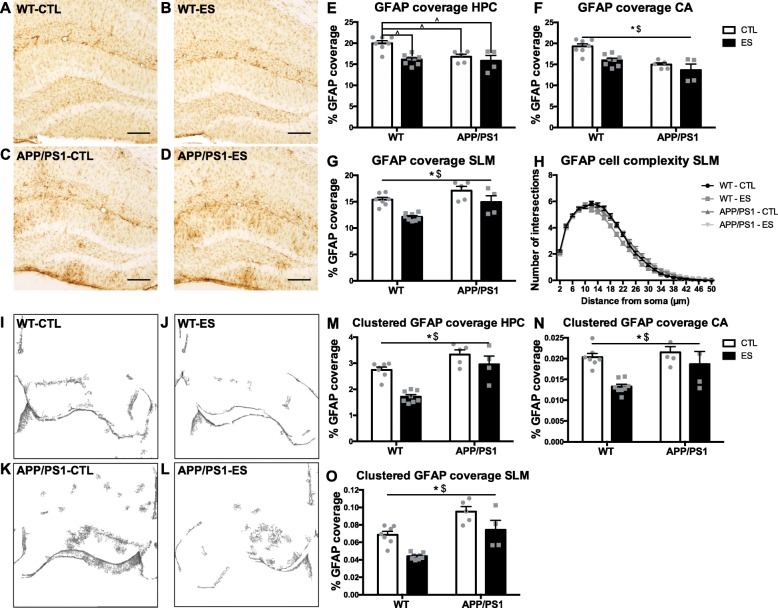
ES and APP/PS1 overexpression affect GFAP coverage in the HPC of 10-month-old mice. Representative images of the HPC of 10-month-old WT-CTL (**a**), WT-ES (**b**), APP/PS1-CTL (**c**), and APP/PS1-ES (**d**) mice. ES modulates the effect of APP/PS1 on GFAP expression in the HPC (**e**). GFAP coverage in the CA was decreased by both ES and APP/PS1 (**f**). APP/PS1 increases hippocampal GFAP expression while ES decreases GFAP expression in the SLM (**g**). GFAP+ cell complexity was unaffected by either ES or APP/PS1 (**h**). Representative images of clustered GFAP coverage in the HPC of 10-month-old WT-CTL (**i**), WT-ES (**j**), APP/PS1-CTL (**k**), and APP/PS1-ES (**l**) mice. Clustered GFAP coverage was increased by APP/PS1 overexpression but decreased by ES in whole HPC (**m**), CA (**n**), and SLM (**o**) region. Statistical analyses were performed using two-way ANOVA. ^$^Significant effect of genotype *p* < 0.05; ^*^Significant effect of condition *p* < 0.05; ^^^Significant post hoc effect *p* < 0.05; scale bars 250 μm

In Fig. 4, example images of GFAP coverage of 10-month-old mice are shown for the HPC of WT-CTL (Fig. 4a), WT-ES (Fig. 4b), APP/PS1-CTL (Fig. 4c), and APP/PS1-ES (Fig. 4d). Clustering of GFAP is observed in APP/PS1 but not WT mice. At 10mo, global GFAP expression in the HPC was affected by both APP/PS1 overexpression and ES (HPC: *F*_condition_(1, 20) = 11.914, *p* = 0.003, *F*_genotype_(1, 20) = 6.419, *p* = 0.020, *F*_condition*genotype_(1, 20) = 4.458, *p* = 0.048 Fig. 4e). Post hoc analyses revealed a significant difference between CTL and ES in WT animals, with GFAP coverage being decreased after ES exposure (WT-CTL–WT-ES: *p* = 0.001 Fig. 4e) but not in APP/PS1 animals (APP/PS1-CTL–APP/PS1-ES: *p* = 832 Fig. 4e). APP/PS1-CTL mice had significantly decreased GFAP coverage compared to WT-CTL mice (WT-CTL–APP/PS1-CTL: *p* = 0.015 Fig. 4e), but this was not the case in ES mice (WT-ES–APP/PS1-ES: *p* = 0.991 Fig. 4e). As for the specific HPC subregions, in the DG, expression of global GFAP was differentially affected by APP/PS1 in ES animals as compared to CTL animals (DG: *F*_condition_(1, 20) = 12.830, *p* = 0.002, *F*_genotype_(1, 20) = 2.499, *p* = 0.130, *F*_condition*genotype_(1, 20) = 10.874, *p* = 0.004). While GFAP expression was unaltered by APP/PS1 in CTL animals (WT-CTL–APP/PS1-CTL: *p* = 0.608), it was increased after ES in transgenic mice (WT-ES–APP/PS1-ES: *p* = 0.015). Also here, ES decreased GFAP coverage in WT (WT-CTL–WT-ES: *p* < 0.001) but not APP/PS1 mice (APP/PS1-CTL–APP/PS1-ES: *p* = 0.998).

In the CA region of the HPC, both ES exposure and APP/PS1 overexpression decreased global GFAP coverage (CA: *F*_condition_(1, 20) = 10.030, *p* = 0.005, *F*_genotype_(1, 20) = 21.457, *p* < 0.001, *F*_condition*genotype_(1, 20) = 1.893, *p* = 0.184 Fig. 4f). Interestingly, when GFAP coverage was separately analyzed in the SLM region of the CA, APP/PS1 increased while ES decreased global GFAP expression (SLM: *F*_condition_(1, 20) = 20.190, *p* < 0.001, *F*_genotype_(1, 20) = 13.812, *p* = 0.001, *F*_condition*genotype_(1, 20) = 0.786, *p* = 0.386 Fig. 4g). Cell complexity analyses showed no differences in either the number of intersections (HPC AUC: *T*_condition_(1, 20) = − 1.526, *p* = 0.143, *T*_genotype_(1, 20) = 0.290, *p* = 0.775, *T*_condition*genotype_(1, 20) = 998, *p* = 0.330 Fig. 4h) or primary processes (HPC: *T*_condition_(1, 20) = − 1.13, *p* = 0.271, *F*_genotype_(1, 20) = − 1.282, *p* = 0.215, *F*_condition*genotype_(1, 20) = 0.939, *p* = 0.359).

Although global GFAP coverage was decreased by APP/PS1, clustering of GFAP immunoreactive signal was observed in the transgenic animals (Fig. 4a–c, d). For this reason, we performed an additional analysis aimed at unraveling the alterations in clustering of GFAP (see Methods). Example pictures of the masking are shown in Fig. 4i–l. We confirm the earlier found decrease in GFAP as induced by ES in the HPC (Fig. 4m). However, clustered GFAP signal was increased in APP/PS1 mice (HPC: *F*_condition_(1, 20) = 19.972, *p* < 0.001, *F*_genotype_(1, 20) = 33.749, *p* < 0.001, *F*_condition*genotype_(1, 20) = 4.288, *p* = 0.052 Fig. 4m). Similar effects were found in the CA (CA: *F*_condition_(1, 20) = 13.734, *p* = 0.001, *F*_genotype_(1, 20) = 5.970, *p* = 0.024, *F*_condition*genotype_(1, 20) = 2.451, *p* = 0.133 Fig. 4n) and SLM (SLM: *F*_condition_(1, 20) = 19.974, *p* < 0.001, *F*_genotype_(1, 20) = 31.041, *p* < 0.001, *F*_condition*genotype_(1, 20) = 0.127, *p* = 0.725 Fig. 4o).

In the DG, expression of GFAP was differentially affected by APP/PS1 in ES animals as compared to CTL animals (DG: *F*_condition_(1, 20) = 12.375, *p* = 0.002, *F*_genotype_(1, 20) = 77.601, *p* < 0.001, *F*_condition*genotype_(1, 20) = 6.695, *p* = 0.018). Further post hoc testing revealed a significant difference between CTL and ES in WT animals, with GFAP coverage being decreased after ES exposure (WT-CTL–WT-ES: *p* < 0.001) but not in the APP/PS1 animals (APP/PS1-CTL–APP/PS1-ES: *p* = 0.935). APP/PS1 significantly increased GFAP coverage in both CTL animals (WT-CTL–APP/PS1-CTL: *p* = 0.001) and ES animals (WT-ES–APP/PS1-ES: *p* < 0.001).

Gene expression levels at 10 months were affected by APP/PS1 overexpression but not by ES (see Table 2). In 10-month-old animals, APP/PS1 overexpression increased mRNA levels of Aqp4, Gfap, and Vimentin, and decreased Aldh1l1.

### The interaction of astrocytes and microglia in ES and amyloid pathology

**Fig. 5:**
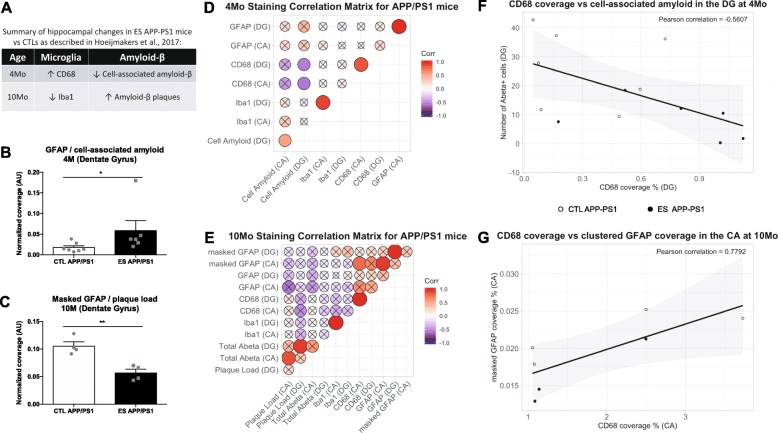
Relationship between astrocytes, microglia, and plaque load in APP/PS1 mice. **a** Summary of microglia and plaque load as published in [14]. **b** Global GFAP is increased in the dentate gyrus (DG) of 4-month-old (4 months) ES-exposed APP/PS1 mice after normalizing to number of amyloid-β + cells. **c** Masked GFAP is decreased in the DG of 10-month-old (10 months) ES-exposed APP/PS1 mice after normalizing to extracellular plaque load. **d**, **e** Pearson correlation coefficients visualized from − 1 (blue) to + 1 (red), with the size of the circles representing the correlation coefficients. Circles without crosses are correlation coefficients significant at *p* < 0.05. **d** Correlation matrix of staining data from 4-month APP/PS1 mice. **e** Correlation matrix between staining data from 10-month APP/PS1 mice. **f** CD68 coverage is negatively correlated with the number of amyloid-beta positive cells in the DG at 4 months. **g** CD68 coverage is positively correlated to masked GFAP coverage in the CA region of 10 months APP/PS1 mice. ^*^Significant effect of condition *p* < 0.05

We have previously described microglial profile and amyloid load in the same cohort of mice used in this study (summarized in Fig. 5a). This allowed us to investigate the link between GFAP coverage and this current set of data. As Aβ protein in APP/PS1 mice is present as cell-associated amyloid at early stages of pathology, and as extracellular plaques at later stages of pathology, we used cell-associated amyloid for 4 months data and plaque load for 10 months data.

Normalized GFAP coverage to cell-associated amyloid is increased in the DG but not CA region in 4 months APP/PS1 mice (DG: Mann-Whitney *U* = 5.0, *p* = 0.0221, Fig. 5b, CA: *t* (11) = 1.532, *p* = 0.1538, not shown). In the 10 months APP/PS1 mice clustered, but not global, GFAP signal in the DG when normalized to extracellular amyloid plaque-load is decreased, (masked GFAP DG: *t* (6) = 4.691, *p* = 0.0034; masked GFAP CA: *t* (6) = 0.4, *p* = 0.703; global GFAP CA: *t* (7) = 0.603, *p* = 0.566; global GFAP DG: *t* (7) = 2.096, *p* = 0.0743). To further explore interactions between the stainings for astrocytes (GFAP), microglia (Iba1, CD68) and Amyloid pathology (6E10), we created correlation matrices of the data derived from the stainings, shown in Fig. 5d (4-month-old APP/PS1 mice) and Fig. 5e (10-month-old APP/PS1 mice). Correlation coefficients and matrices were also calculated and created for WT mice (Supplementary Fig. 1).

#### Interaction of GFAP, amyloid pathology, and microglia at 4 months

Amyloid pathology did not correlate with GFAP (CA: *r* = 0.211, *p* = 0.469; DG: *r* = 0.422, *p* = 0.133) or Iba1 (CA: *r* = 0.328, *p* = 0.215; DG: *r* = − 0.055, *p* = 0.841) expression in the HPC. In contrast, the number of Aβ-positive cells at 4 months was negatively correlated to expression of the microglial phagocytic marker CD68 (DG: *r* = − 0.561, *p* = 0.046, Fig. 5f; CA: *r* = − 0.4594, *p* = 0.099). When exploring the relationship between astrocyte and microglial markers in the HPC in WT and APP/PS1 mice, while we do not find significant correlations between astrocytes and microglial markers in the HPC, the magnitude of the correlation coefficients in WT mice (Supplementary Fig. 1) was much stronger than in APP/PS1 mice. This was the case for both the CA (WT GFAP vs Iba1: *r* = 0.376, *p* = 0.124; WT GFAP vs CD68: *r* = 0.504, *p* = 0.056; APP/PS1 GFAP vs Iba1: *r* = 0.151, *p* = 0.606; APP/PS1 GFAP vs CD68: *r* = 0.022, *p* = 0.947) and the DG (WT GFAP vs Iba1: *r* = 0.3910, *p* = 0.109; WT GFAP vs CD68: *r* = 0.414, *p* = 0.125; APP/PS1 GFAP vs Iba1: *r* = − 0.116, *p* = 0.694; APP/PS1 GFAP vs CD68: *r* = 0.153, *p* = 0.655).

#### Interaction of GFAP, amyloid pathology, and microglia at 10 months

Plaque load did not significantly correlate with Iba1 (CA: *r* = 0.043, *p* = 0.906; DG: − 0.283, *p* = 0.428), CD68 (CA: *r* = 0.170, *p* = 0.687; DG: − 0.398, *p* = 0.329), or GFAP (CA: *r* = − 0.599, *p* = 0.089; DG: − 0.064, *p* = 0.870). Similarly, there was no correlation at this age between total Aβ and either Iba1 (CA: *r* = 0.073, *p* = 0.841; DG: − 0.119, *p* = 0.743), CD68 (CA: *r* = 0.084, *p* = 0.843; DG: *r* = − 0.166, *p* = 0.694) or GFAP (CA: *r* = − 0.473, *p* = 0.198; DG: − 0.085, *p* = 0.827). Regarding the relationship between astrocytes and microglia, hippocampal GFAP expression was not significantly correlated with Iba1 expression in 10-month-old WT (CA: *r* = 0.140, *p* = 0.620; DG: 0.311, *p* = 0.260) or APP/PS1 animals (CA: *r* = − 0.184, *p* = 0.636; DG: 0.080, *p* = 0.839). There was similarly no correlation between GFAP expression and CD68 coverage in WT (CA: *r* = 0.331, *p* = 0.248; DG: 0.230, *p* = 0.428) or APP/PS1 (CA: *r* = 0.640, *p* = 0.122; DG: 0.235, *p* = 0.612) animals, although there was a significant correlation in the CA region of APP/PS1 animals between CD68 and masked GFAP coverage (CA: *r* = 0.779, *p* = 0.039, Fig. 5g; DG: 0.216, *p* = 0.642). Similar to the 4-month data, there were several strong positive correlations between these proteins between hippocampal subregions in both WT (not shown) and APP/PS1 (Fig. 5e) mice, although plaque load (*r* = 0.218, *p* = 0.545) and GFAP (GFAP: *r* = 0.393, *p* = 0.296; masked GFAP: *r* = 0.320, *p* = 0.401) of APP/PS1 mice were not correlated between the CA and DG. GFAP was correlated in WT animals between both subregions (GFAP: *r* = 0.885, *p* < 0.001; masked GFAP: *r* = 0.860, *p* < 0.001).

## Discussion

**Fig. 6:**
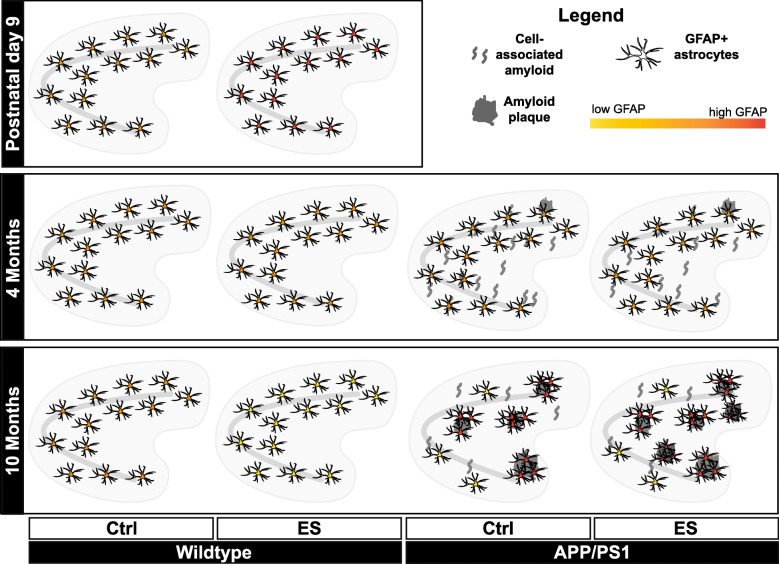
Overview of ES and APP/PS1-induced effects on hippocampal astrocytic GFAP. Stress exposure early in life from postnatal days 2–9 is associated with an increase of GFAP in specific hippocampal subregions at P9. No effects are observed at 4 months. At 10 months, GFAP coverage is reduced in wildtype mice. In APP/PS1 mice, no effects are observed at 4 months (early pathological stage) of either genotype or ES exposure. At 10 months (advanced pathological stage), we observe genotype-induced reduction in global and increase in localized GFAP clustering, presumably due to Aβ accumulation. Notably, amyloid pathology is modulated by ES [14], decreasing cell-associated amyloid at 4 months, and increasing amyloid load at 10 months. While ES did not affect the absolute measures of GFAP, this suggests altered astrocytic response to amyloid pathology after ES

Here, we studied how ES affects astrocytes at different ages and whether ES alters the astrocytic response to Aβ neuropathology at early- and advanced-pathological stages in APP/PS1 mice. At P9, an increase in GFAP expression was found only in the SLM region of the HPC, without any further changes at the age of P30, 4, and 6 months. At 10 months however, we found that ES leads to a significant reduction of global hippocampal GFAP coverage. Morphologically, APP/PS1 10-month mice showed both “isolated” as well as “clustered” forms of GFAP, which were separable when processing by size. APP/PS1 mice at 10 months exhibited an increased global (isolated and clustered) coverage of GFAP in the EC, decreased global coverage of GFAP in the HPC, as well as increased clustered GFAP in both regions. APP/PS1 overexpression also led to altered expression of astrocyte-related genes in the HPC at this advanced pathological stage, which was not further modulated by ES exposure. Notably, when analyzed as a function of amyloid load, we find GFAP in ES-exposed mice increased at 4 months and reduced at 10 months. Finally, based on the correlation matrices, coverage of clustered GFAP expression correlated to CD68 expression in the CA of 10-month APP/PS1 mice, while no other correlations were detected between GFAP, microglial markers, or plaque pathology at 4 or 10 months. The findings related to the hippocampus are summarized in Fig. 6. We will discuss our findings first in the context of the ES exposure and wildtype mice and thereafter in the context of APP/PS1 mice.

### Modulation of astrocytes by ES in wildtype mice across lifespan

To our knowledge, this is the first study addressing effects of ES on GFAP expression ES at multiple different ages across the lifespan in the HPC using mice and the limited nesting model. The effects of ES on GFAP expression in WT mice are age and brain region dependent. At P9, a significant increase in GFAP immunoreactivity was present following ES exposure specifically in the SLM but not in other hippocampal subregions. Strikingly, the existing literature shows that ES in the form of maternal deprivation or separation leads to an acute reduction in GFAP [36, 37, 57, 58], followed by an increase in GFAP when a longer period (4–30 days) between the stressor and the analysis was applied [38, 59, 60]. This discrepancy with our current results could possibly be explained by the form of ES exposure. Notably, the limited nesting and bedding ES model applied in this study have a more chronic nature, as it is a continuous exposure to an impoverished environment for 7 days, versus a single episode or intermittent exposure in maternal deprivation and separation models respectively. Thus, the differences in intensity and length of exposure could determine the final effect on astrocytes.

When looking at more lasting effects of ES exposure, GFAP expression in the HPC appeared unaffected at 4 and 6 months of age, while reduced in 10-month-old mice exposed to ES. This finding is in line with the only other report showing that 12 months old rats exposed to maternal deprivation exhibit reduced GFAP [35]. Together, these results suggest that ES programming of astrocytes might only become apparent during aging. Intriguingly, our data suggest that the response of GFAP expression to ES exposure changes over time from an acute upregulation in a specific hippocampal subregion to a long-term downregulation. Such temporal dynamics of GFAP expression, even though on a shorter timescale, have also been observed previously in rats exposed to daily maternal deprivation from P1 to P10. In these rats, no initial changes were observed at P10, but a reduction in GFAP was observed at P20, followed by increased GFAP at P40 [60]. This direction of GFAP modulation at the different ages is not in line with the changes observed in the present study. This could be related to differences in frequency and intensity of the ES model, as well as potential species-specific effects, and further stresses the relevance of the specifics of the ES model. Despite the discussed discrepancies, both studies suggest complex temporal dynamics of GFAP expression in response to ES and show that the effect of ES on GFAP expression is not only lasting but also depends on the neurodevelopmental or lifetime stage.

When examining the specific characteristics of GFAP leading to the modulation of coverage, notably, no alterations in the number or complexity of GFAP+ cells were found as a consequence of ES in any of the age groups. This indicates that ES-induced alterations in GFAP result from intracellular GFAP changes, rather than a change in general astrocyte numbers or complexity [52, 61]. For the full interpretation of our findings, it is important to note that GFAP does not label all astrocytes in the brain, although a large part of astrocytes in the hippocampus is GFAP-positive [62]. Additionally, ES-induced changes in astrocytes can also occur in the absence of GFAP alterations [63]. Next to GFAP protein levels, gene expression analyses showed that ES did not affect gene expression levels of astrocyte-related genes (i.e., Aldh1l1, Aqp4, Glast, Glt1, GluS, Gfap, Vimentin) at P9, 4, or 10 months, with the exception of Fasn mRNA levels, which were increased by ES at 4 months. Fasn is a key enzyme for de novo lipogenesis [64], a process in which astrocytes are highly active [27, 65, 66]. Changes in Fasn gene expression have been implicated in hippocampal changes in neurogenesis [67] and could indicate disrupted lipid metabolism as a result of ES, which might have implications for synapse development [27]. However, further investigations will need to clarify what the functional consequences of this change might be.

### Modulation of astrocytes by Aβ overexpression in HPC and EC

APP/PS1 overexpression in mice did not lead to alterations in astrocytes at early-pathological stages but did alter GFAP expression in the HPC and EC, as well as the HPC expression of several astrocyte-related genes, in 10-month-old-mice. In AD patients, GFAP+ reactive astrocytes are particularly associated with Aβ plaques (i.e., astrogliosis) [18, 20–22]. The lack of astrogliosis in the HPC and EC at 4 months is thus in line with the fact that only few Aβ plaques are present during this early-pathological stage [14].

The EC presents with the earliest signs of Aβ pathology in patients [68]. Here, we presented that GFAP immunoreactivity and GFAP cell complexity in the EC was increased in 10-month-old APP/PS1 mice. Similarly, injections with Aβ peptide in the EC caused increased GFAP intensity and larger GFAP cell somas [23], streptozotocin-induced AD in rats resulted in increased numbers of GFAP+ cells in the EC [24], and numbers of GFAP+ cells were increased in the EC of APP/Tau transgenic mice [69]. In contrast, it was reported that astrocytes in the EC appeared atrophied in a triple transgenic AD mouse model (3xTG), an effect that became less pronounced at a more advanced pathological stage (9 months) [70]. In this study, the use of a different transgenic line possibly explains the discrepancy with our current findings, as e.g., age-related epigenetic changes differ between different mouse models of AD [71]. In addition, it is important to note that more research points towards a heterogeneous response of astrocytes in response to AD pathology, as both astrocyte reactivity and atrophy occurs in AD (reviewed in [72]).

In contrast to the EC, we observed a global decrease in GFAP expression in the HPC, while most research demonstrates increased GFAP coverage in the HPC of APP/PS1 mice ranging from 5 to 14 months of age [73–75]. However, importantly, clustering of GFAP was observed both in the EC and HPC of 10-month-old APP/PS1 mice, and coverage of this clustered GFAP in the HPC was increased as expected. Considering that clustered GFAP was more prominent in APP/PS1 mice, and we observed a similar pattern of Aβ plaque load in parallel series of the same brain stained for Aβ, clustering of GFAP is probably due to accumulation of GFAP around Aβ plaques [76]. The reduction in global versus the increase in clustered GFAP coverage is in line with a previous study where general astroglial cytoskeletal atrophy was observed, while GFAP+ astrocytes surrounding Aβ plaques were hypertrophic in the HPC of 18-month-old 3xTG AD mice [77]. Given that astrocytes do not migrate to amyloid plaques [78], current data suggests that astrocytes near plaques are induced to upregulate GFAP expression, while astrocytes in the rest of the HPC downregulate GFAP. As many extracellular factors, e.g., inflammatory factors, can affect transcription and translation of GFAP [79, 80], microenvironmental changes induced by plaque accumulation may lead to local dynamic alterations in GFAP expression.

Gene expression analyses revealed that Gfap and Vimentin mRNA expression was not altered at 4 but increased at 10 months old. A similar increase in Gfap mRNA was previously shown in AD mice [74] and is in line with the appearance of reactive astrocytes [81]. While the exact function of reactive astrocytes is still not fully understood, genetic deletion of Gfap and Vimentin increased plaque load in APP/PS1 mice [82], suggesting that acquiring a reactive phenotype supports astrocytes in limiting amyloid pathology. Interestingly, Gfap mRNA in a full hippocampal homogenate was increased while we observed decreased global hippocampal GFAP protein coverage and increased clustered GFAP coverage. This suggests that, while indeed overall GFAP levels seem associated with plaque load and Aβ probably drives astrogliosis [83, 84], GFAP dynamics might be differentially affected by the microenvironment based on proximity to plaques. Aqp4 mRNA expression was also increased in APP/PS1 mice at 10 months. Aqp4 is the major water channel expressed in the CNS, and its expression is mostly restricted to astrocytes. Accumulating evidence suggests that Aqp4 plays a role in AD pathology [85] and the glymphatic system [86, 87] and may mediate clearance of Aβ [85, 88]. In line with our findings, multiple studies have found increased levels of Aqp4 in AD patients [89–91]. Furthermore, a decrease in Aldh1l1 was present in APP/PS1 mice. The decrease in Aldh1l1 could suggest a global loss of astrocytes, however, previous research revealed no difference in cortical astrocyte number between healthy and AD brains [92, 93], suggesting that this is probably not the case. Further research is necessary to understand the implication of the reduction of Aldh1l1.

### Modulation of astrocytes by ES in Aβ overexpressing mice

At 10 months, both ES and APP/PS1 overexpression independently decreased global GFAP levels in the HPC. We see in the CTL APP/PS1 mice a distinction between global and clustered GFAP signal, and our analyses show that the two can be disentangled when accounting for aggregation of GFAP signals. Interestingly, neither the global nor the clustered signal is further affected by ES. However, we know from our previous study in the same cohort of mice that ES exposure had age-dependent effects on hippocampal amyloid load, and we in fact find ES-induced differences in GFAP coverage when normalizing to the appropriate pathology readout (Fig. 5a–c). Thus, it seems that ES exposure might still lead to some latent differential astroglial response to Aβ. This would be in line with decreased microglial accumulation observed in this cohort [14].

There is now a growing body of literature showing that astrocytes and microglia interact. Microglia are known to activate astrocytes during neuroinflammation [94, 95] and astrocytes can influence microglial activity, e.g., by releasing factors that increase phagocytosis [96]. Furthermore, emerging evidence is available for the synergistic effect of astrocytes and microglia in the progression of AD pathology [97, 98]. At 4 months, cell-associated amyloid is not correlated to either GFAP or Iba1 but negatively correlates to the microglial phagocytic marker CD68. This is in line with the fact that at this stage mice display mostly cell-associated amyloid-β and only few extracellular plaques; thus, no strong increase in activated microglia or astrogliosis would be expected. Still, Aβ, even in its cell-associated form, is considered somewhat inflammatory [99], and the negative correlation between cell-associated amyloid and CD68 further supports that the earlier reported ES-induced increase in phagocytic microglia might contribute to the reduction in cell-associated amyloid in APP/PS1 mice exposed to ES [14].

At 10 months, we find no correlations between plaque load and total amyloid with GFAP, Iba1, or CD68. This could be due to a saturated response, i.e., as Aβ continues to accumulate, the response of the glial cells does not parallel the increase in plaques. This is in line with the idea that neuroinflammation in AD shifts from being driven to respond to pathology (e.g., via initial Aβ buildup) to being a driving, self-perpetuating factor in disease progression due to the chronic neuroinflammatory environment it has created [100]. Interestingly, at 10 months, plaque load in the DG and CA do not correlate. Similarly, we did not detect correlations in APP/PS1 mice of global and clustered GFAP coverage between the two subregions, supporting the notion that amyloid pathology is changing the dynamics of GFAP expression within the HPC and leading to localized astrocytic responses that might not be reflected in a global analysis. When researching the relationship between astrocyte and microglia in AD pathology, we detected a significant relationship between CD68 and clustered GFAP coverage in the CA region. This might partly reflect the response of both CD68 and clustered GFAP signal to amyloid plaque buildup, perhaps pointing towards coordination of microglial and astrocytic phagocytosis, as reactive astrocytes are also capable of internalizing Aβ [101]. There were no other correlations detected between GFAP and Iba1 or CD68 in 10-month-old mice. More studies are necessary to unravel how precisely these two cell types interact in the response to Aβ pathology.

## Conclusion

In summary, we have shown that ES age-dependently affects GFAP protein expression over the course of a lifetime. Further analyses at the level of gene expression revealed minimal ES effects on astrocyte-related genes at any of the timepoints studied, except for FASN at 4 months. In APP/PS1 mice, we confirm expected amyloid-induced changes to astrocyte gene expression and GFAP signal at different pathological stages.

While these effects were not further modulated by ES, effects of ES exposure appear when normalizing GFAP coverage to ES-induced alterations to amyloid pathology [14], suggesting a differential astrocytic response to this pathology. Further research is needed to elucidate whether local interactions might be occurring.

## Supplementary information

**Additional file 1: Figure S1.** Relationship between astrocytes and microglia in wild type mice. (**A-B**) Pearson correlation coefficients visualized from − 1 (blue) to + 1 (red), with the size of the circles representing the correlation coefficients. Circles without crosses are correlation coefficients significant at *p* < 0.05. (**A**) Correlation matrix of staining data from 4mo WT mice. (**B**) Correlation matrix between staining data from 10mo WT mice. (EPS 3753 kb)

## Data Availability

The data that support the findings of this study are available from the corresponding author upon reasonable request.

## Abbreviations

AD: Alzheimer’s disease
Aβ: Amyloid-beta
CNS: Central nervous system
CTL: Control
ES: Early-life stress
GFAP: Glial fibrillary acidic protein
P: Postnatal day

## References

[1] Pesonen Anu-Katriina, Eriksson Johan G., Heinonen Kati, Kajantie Eero, Tuovinen Soile, Alastalo Hanna, Henriksson Markus, Leskinen Jukka, Osmond Clive, Barker David J.P., Räikkönen Katri (2013). Cognitive ability and decline after early life stress exposure. Neurobiology of Aging.

[2] Pechtel Pia, Pizzagalli Diego A. (2010). Effects of early life stress on cognitive and affective function: an integrated review of human literature. Psychopharmacology.

[3] Saleh A, Potter GG, McQuoid DR, Boyd B, Turner R, MacFall JR (2017). Effects of early life stress on depression, cognitive performance and brain morphology. Psychol Med.

[4] Hedges DW, Woon FL (2010). Early-life stress and cognitive outcome. Psychopharmacology.

[5] Hoeijmakers L, Lesuis SL, Krugers H, Lucassen PJ, Korosi A (2018). A preclinical perspective on the enhanced vulnerability to Alzheimer’s disease after early-life stress. Neurobiol Stress.

[6] Lesuis SL, Hoeijmakers L, Korosi A, De Rooij SR, Swaab DF, Kessels HW (2018). Vulnerability and resilience to Alzheimer’s disease: early life conditions modulate neuropathology and determine cognitive reserve. Alzheimers Res Ther.

[7] Wang Lan, Yang Linlin, Yu Lulu, Song Mei, Zhao Xiaochuan, Gao Yuanyuan, Han Keyan, An Cuixia, Xu Shunjiang, Wang Xueyi (2016). Childhood physical neglect promotes development of mild cognitive impairment in old age – A case-control study. Psychiatry Research.

[8] Ravona-Springer R, Beeri MS, Goldbourt U (2012). Younger age at crisis following parental death in male children and adolescents is associated with higher risk for dementia at old age. Alzheimer Dis Assoc Disord.

[9] Norton MC, Smith KR, Østbye T, Tschanz JT, Schwartz S, Corcoran C (2011). Early parental death and remarriage of widowed parents as risk factors for Alzheimer disease. Am J Geriatr Psychiatry.

[10] Donley Gwendolyn A R, Lönnroos Eija, Tuomainen Tomi-Pekka, Kauhanen Jussi (2018). Association of childhood stress with late-life dementia and Alzheimer’s disease: the KIHD study. European Journal of Public Health.

[11] Heneka Michael T, Carson Monica J, Khoury Joseph El, Landreth Gary E, Brosseron Frederic, Feinstein Douglas L, Jacobs Andreas H, Wyss-Coray Tony, Vitorica Javier, Ransohoff Richard M, Herrup Karl, Frautschy Sally A, Finsen Bente, Brown Guy C, Verkhratsky Alexei, Yamanaka Koji, Koistinaho Jari, Latz Eicke, Halle Annett, Petzold Gabor C, Town Terrence, Morgan Dave, Shinohara Mari L, Perry V Hugh, Holmes Clive, Bazan Nicolas G, Brooks David J, Hunot Stéphane, Joseph Bertrand, Deigendesch Nikolaus, Garaschuk Olga, Boddeke Erik, Dinarello Charles A, Breitner John C, Cole Greg M, Golenbock Douglas T, Kummer Markus P (2015). Neuroinflammation in Alzheimer's disease. The Lancet Neurology.

[12] Jafari Z, Okuma M, Karem H, Mehla J, Kolb BE, Mohajerani MH (2019). Prenatal noise stress aggravates cognitive decline and the onset and progression of beta amyloid pathology in a mouse model of Alzheimer’s disease. Neurobiol Aging.

[13] Hui Jianjun, Feng Gaifeng, Zheng Caifeng, Jin Hui, Jia Ning (2017). Maternal separation exacerbates Alzheimer’s disease-like behavioral and pathological changes in adult APPswe/PS1dE9 mice. Behavioural Brain Research.

[14] Hoeijmakers Lianne, Ruigrok Silvie R., Amelianchik Anna, Ivan Daniela, van Dam Anne-Marie, Lucassen Paul J., Korosi Aniko (2017). Early-life stress lastingly alters the neuroinflammatory response to amyloid pathology in an Alzheimer’s disease mouse model. Brain, Behavior, and Immunity.

[15] Lesuis SL, Maurin H, Borghgraef P, Lucassen PJ, Van Leuven F, Krugers HJ, et al. Positive and negative early life experiences differentially modulate long term survival and amyloid protein levels in a mouse model of Alzheimer’s disease. Oncotarget. 2016;7:39118–35. 10.18632/oncotarget.9776 PMC5129918 27259247

[16] Lesuis SL, Kaplick PM, Lucassen PJ, Krugers HJ (2019). Treatment with the glutamate modulator riluzole prevents early life stress-induced cognitive deficits and impairments in synaptic plasticity in APPswe/PS1dE9 mice. Neuropharmacology..

[17] Hoeijmakers L, Amelianchik A, Verhaag F, Kotah J, Lucassen PJ, Korosi A (2018). Early-life stress does not aggravate spatial memory or the process of hippocampal neurogenesis in adult and middle-aged APP/PS1 mice. Front Aging Neurosci.

[18] Kamphuis W, Middeldorp J, Kooijman L, Sluijs JA, Kooi E-J, Moeton M (2014). Glial fibrillary acidic protein isoform expression in plaque related astrogliosis in Alzheimer’s disease. Neurobiol Aging.

[19] Pekny M, Pekna M (2014). Astrocyte reactivity and reactive astrogliosis: costs and benefits. Physiol Rev.

[20] Kato S, Gondo T, Hoshii Y, Takahashi M, Yamada M, Ishihara T (1998). Confocal observation of senile plaques in Alzheimer’s disease: senile plaque morphology and relationship between senile plaques and astrocytes. Pathol Int.

[21] Osborn LM, Kamphuis W, Wadman WJ, Hol EM (2016). Astrogliosis: an integral player in the pathogenesis of Alzheimer’s disease. Prog Neurobiol.

[22] Vijayan VK, Gedes JW, Anderson KJ, Chang-Chui H, Ellis WG, Cotman CW (1991). Astrocyte hypertrophy in the Alzheimer’s disease hippocampal formation. Exp Neurol.

[23] Sipos E, Kurunczi A, Kasza Á, Horváth J, Felszeghy K, Laroche S (2007). β-Amyloid pathology in the entorhinal cortex of rats induces memory deficits: implications for Alzheimer’s disease. Neuroscience..

[24] Chen Yanxing, Guo Zhangyu, Mao Yan-Fang, Zheng Tingting, Zhang Baorong (2017). Intranasal Insulin Ameliorates Cerebral Hypometabolism, Neuronal Loss, and Astrogliosis in Streptozotocin-Induced Alzheimer’s Rat Model. Neurotoxicity Research.

[25] Allen NJ (2014). Astrocyte regulation of synaptic behavior. Annu Rev Cell Dev Biol.

[26] Allen NJ, Eroglu C (2017). Cell biology of astrocyte-synapse interactions. Neuron..

[27] van Deijk Anne-Lieke F., Camargo Nutabi, Timmerman Jaap, Heistek Tim, Brouwers Jos F., Mogavero Floriana, Mansvelder Huibert D., Smit August B., Verheijen Mark H.G. (2017). Astrocyte lipid metabolism is critical for synapse development and function in vivo. Glia.

[28] Masliah E, Mallory M, Alford M, DeTeresa R, Hansen LA, McKeel DW (2001). Altered expression of synaptic proteins occurs early during progression of Alzheimer’s disease. Neurology..

[29] Scheff SW, Price DA, Schmitt FA, Mufson EJ (2006). Hippocampal synaptic loss in early Alzheimer’s disease and mild cognitive impairment. Neurobiol Aging.

[30] Scheff SW, Price DA (1993). Synapse loss in the temporal lobe in Alzheimer’s disease. Ann Neurol.

[31] Forner S, Baglietto-Vargas D, Martini AC, Trujillo-Estrada L, LaFerla FM (2017). Synaptic impairment in Alzheimer’s disease: a dysregulated symphony. Trends Neurosci.

[32] Terry RD, Masliah E, Salmon DP, Butters N, DeTeresa R, Hill R (1991). Physical basis of cognitive alterations in Alzheimer’s disease: synapse loss is the major correlate of cognitive impairment. Ann Neurol.

[33] Abbink MR, van Deijk A-LF, Heine VM, Verheijen MH, Korosi A (2019). The involvement of astrocytes in early-life adversity induced programming of the brain. Glia..

[34] Gunn B. G., Cunningham L., Cooper M. A., Corteen N. L., Seifi M., Swinny J. D., Lambert J. J., Belelli D. (2013). Dysfunctional Astrocytic and Synaptic Regulation of Hypothalamic Glutamatergic Transmission in a Mouse Model of Early-Life Adversity: Relevance to Neurosteroids and Programming of the Stress Response. Journal of Neuroscience.

[35] Leventopoulos M, Rüedi-Bettschen D, Knuesel I, Feldon J, Pryce CR, Opacka-Juffry J (2007). Long-term effects of early life deprivation on brain glia in Fischer rats. Brain Res.

[36] Saavedra LM, Fenton Navarro B, Torner L (2017). Early life stress activates glial cells in the hippocampus but attenuates cytokine secretion in response to an immune challenge in rat pups. Neuroimmunomodulation..

[37] Roque A, Ochoa-Zarzosa A, Torner L (2016). Maternal separation activates microglial cells and induces an inflammatory response in the hippocampus of male rat pups, independently of hypothalamic and peripheral cytokine levels. Brain Behav Immun.

[38] Llorente Ricardo, Gallardo Meritxell López, Berzal Alvaro Llorente, Prada Carmen, Garcia‐Segura Luis Miguel, Viveros María‐Paz (2009). Early maternal deprivation in rats induces gender‐dependent effects on developing hippocampal and cerebellar cells. International Journal of Developmental Neuroscience.

[39] Yin Zhuoran, Raj Divya, Saiepour Nasrin, Van Dam Debby, Brouwer Nieske, Holtman Inge R., Eggen Bart J.L., Möller Thomas, Tamm Joseph A., Abdourahman Aicha, Hol Elly M., Kamphuis Willem, Bayer Thomas A., De Deyn Peter P., Boddeke Erik (2017). Immune hyperreactivity of Aβ plaque-associated microglia in Alzheimer's disease. Neurobiology of Aging.

[40] Bouvier DS, Jones EV, Quesseveur G, Davoli MA, Ferreira TA, Quirion R (2016). High resolution dissection of reactive glial nets in Alzheimer’s disease. Sci Rep.

[41] Dong Y, Benveniste EN (2001). Immune function of astrocytes. Glia..

[42] Farina C, Aloisi F, Meinl E (2007). Astrocytes are active players in cerebral innate immunity. Trends Immunol.

[43] Jha MK, Jo M, Kim JH, Suk K (2019). Microglia-astrocyte crosstalk: an intimate molecular conversation. Neuroscientist..

[44] Apelt J, Schliebs R (2001). Beta-amyloid-induced glial expression of both pro- and anti-inflammatory cytokines in cerebral cortex of aged transgenic Tg2576 mice with Alzheimer plaque pathology. Brain Res.

[45] Orre Marie, Kamphuis Willem, Osborn Lana M., Jansen Anne H.P., Kooijman Lieneke, Bossers Koen, Hol Elly M. (2014). Isolation of glia from Alzheimer's mice reveals inflammation and dysfunction. Neurobiology of Aging.

[46] Savonenko A, Xu GM, Melnikova T, Morton JL, Gonzales V, Wong MPF (2005). Episodic-like memory deficits in the APPswe/PS1dE9 mouse model of Alzheimer’s disease: relationships to β-amyloid deposition and neurotransmitter abnormalities. Neurobiol Dis.

[47] Jankowsky Joanna L, Slunt Hilda H, Ratovitski Tamara, Jenkins Nancy A, Copeland Neal G, Borchelt David R (2001). Co-expression of multiple transgenes in mouse CNS: a comparison of strategies. Biomolecular Engineering.

[48] Naninck Eva F.G., Hoeijmakers Lianne, Kakava-Georgiadou Nefeli, Meesters Astrid, Lazic Stanley E., Lucassen Paul J., Korosi Aniko (2014). Chronic early life stress alters developmental and adult neurogenesis and impairs cognitive function in mice. Hippocampus.

[49] Derveaux S, Vandesompele J, Hellemans J (2010). How to do successful gene expression analysis using real-time PCR. Methods..

[50] Hellemans J, Mortier G, De Paepe A, Speleman F, Vandesompele J (2007). qBase relative quantification framework and software for management and automated analysis of real-time quantitative PCR data. Genome Biol.

[51] Kang K, Lee S-W, Han JE, Choi JW, Song M-R (2014). The complex morphology of reactive astrocytes controlled by fibroblast growth factor signaling. Glia..

[52] Tynan Ross J., Beynon Sarah B., Hinwood Madeleine, Johnson Sarah J., Nilsson Michael, Woods Jason J., Walker Frederick R. (2013). Chronic stress-induced disruption of the astrocyte network is driven by structural atrophy and not loss of astrocytes. Acta Neuropathologica.

[53] Team RC (2018). A language and environment for statistical computing.

[54] Pinheiro J, Bates D, DebRoy S, Sarkar D, R Core Team (2019). nlme: linear and nonlinear mixed effects models.

[55] Kassambara A (2018). ggcorrplot: visualization of a correlation matrix using’ggplot2’.

[56] Wickham H (2016). ggplot2: elegant graphics for data analysis.

[57] Musholt Kristina, Cirillo Giovanni, Cavaliere Carlo, Rosaria Bianco Maria, Bock Joerg, Helmeke Carina, Braun Katharina, Papa Michele (2009). Neonatal separation stress reduces glial fibrillary acidic protein- and S100β-immunoreactive astrocytes in the rat medial precentral cortex. Developmental Neurobiology.

[58] Braun K, Antemano R, Helmeke C, Büchner M, Poeggel G (2009). Juvenile separation stress induces rapid region- and layer-specific changes in S100ß- and glial fibrillary acidic protein-immunoreactivity in astrocytes of the rodent medial prefrontal cortex. Neuroscience..

[59] Kwak HR, Lee JW, Kwon K-J, Kang CD, Cheong IY, Chun W (2009). Maternal social separation of adolescent rats induces hyperactivity and anxiolytic behavior. Korean J Physiol Pharmacol.

[60] Réus Gislaine Z., Silva Ritele H., de Moura Airam B., Presa Jaqueline F., Abelaira Helena M., Abatti Mariane, Vieira Andriele, Pescador Bruna, Michels Monique, Ignácio Zuleide M., Dal-Pizzol Felipe, Quevedo João (2018). Early Maternal Deprivation Induces Microglial Activation, Alters Glial Fibrillary Acidic Protein Immunoreactivity and Indoleamine 2,3-Dioxygenase during the Development of Offspring Rats. Molecular Neurobiology.

[61] Wilhelmsson U, Bushong EA, Price DL, Smarr BL, Phung V, Terada M (2006). Redefining the concept of reactive astrocytes as cells that remain within their unique domains upon reaction to injury. Proc Natl Acad Sci U S A.

[62] Khakh BS, Sofroniew MV (2015). Diversity of astrocyte functions and phenotypes in neural circuits. Nat Neurosci.

[63] Gosselin R-D, O’Connor RM, Tramullas M, Julio-Pieper M, Dinan TG, Cryan JF (2010). Riluzole normalizes early-life stress-induced visceral hypersensitivity in rats: role of spinal glutamate reuptake mechanisms. Gastroenterology..

[64] Ameer F, Scandiuzzi L, Hasnain S, Kalbacher H, Zaidi N (2014). De novo lipogenesis in health and disease. Metabolism..

[65] Camargo N, Brouwers JF, Loos M, Gutmann DH, Smit AB, Verheijen MHG (2012). High-fat diet ameliorates neurological deficits caused by defective astrocyte lipid metabolism. FASEB J.

[66] Hofmann K, Rodriguez-Rodriguez R, Gaebler A, Casals N, Scheller A, Kuerschner L. Astrocytes and oligodendrocytes in grey and white matter regions of the brain metabolize fatty acids. Sci Rep. 2017;7:10779. 10.1038/s41598-017-11103-5 PMC5589817 28883484

[67] Knobloch M, Braun SMG, Zurkirchen L, von Schoultz C, Zamboni N, Araúzo-Bravo MJ (2013). Metabolic control of adult neural stem cell activity by Fasn-dependent lipogenesis. Nature..

[68] Zhou M, Zhang F, Zhao L, Qian J, Dong C (2016). Entorhinal cortex: a good biomarker of mild cognitive impairment and mild Alzheimer’s disease. Rev Neurosci.

[69] DaRocha-Souto B, Scotton TC, Coma M, Serrano-Pozo A, Hashimoto T, Serenó L (2011). Brain oligomeric $β$-amyloid but not total amyloid plaque burden correlates with neuronal loss and astrocyte inflammatory response in amyloid precursor protein/tau transgenic mice. J Neuropathol Exp Neurol.

[70] Yeh C-Y, Vadhwana B, Verkhratsky A, Rodriguez JJ (2011). Early astrocytic atrophy in the entorhinal cortex of a triple transgenic animal model of Alzheimer’s disease. ASN Neuro.

[71] Lardenoije R, van den Hove DLA, Havermans M, van Casteren A, Le KX, Palmour R (2018). Age-related epigenetic changes in hippocampal subregions of four animal models of Alzheimer’s disease. Mol Cell Neurosci.

[72] Rodríguez JJ, Butt AM, Gardenal E, Parpura V, Verkhratsky A (2016). Complex and differential glial responses in Alzheimers disease and ageing. Curr Alzheimer Res..

[73] Zhu S, Wang J, Zhang Y, He J, Kong J, Wang J-F (2017). The role of neuroinflammation and amyloid in cognitive impairment in an APP/PS1 transgenic mouse model of Alzheimer’s disease. CNS Neurosci Ther.

[74] McDonald CL, Hennessy E, Rubio-Araiz A, Keogh B, McCormack W, McGuirk P (2016). Inhibiting TLR2 activation attenuates amyloid accumulation and glial activation in a mouse model of Alzheimer’s disease. Brain Behav Immun.

[75] Shi Xiao-meng, Zhang Hua, Zhou Zhang-jiuzhi, Ruan Ying-ying, Pang Jie, Zhang Lu, Zhai Wei, Hu Yan-li (2018). Effects of safflower yellow on beta-amyloid deposition and activation of astrocytes in the brain of APP/PS1 transgenic mice. Biomedicine & Pharmacotherapy.

[76] Olsen M, Aguilar X, Sehlin D, Fang XT, Antoni G, Erlandsson A (2018). Astroglial responses to amyloid-beta progression in a mouse model of Alzheimer’s disease. Mol Imaging Biol.

[77] Olabarria M, Noristani HN, Verkhratsky A, Rodríguez JJ (2010). Concomitant astroglial atrophy and astrogliosis in a triple transgenic animal model of Alzheimer’s disease. Glia..

[78] Galea E, Morrison W, Hudry E, Arbel-Ornath M, Bacskai BJ, Gómez-Isla T (2015). Topological analyses in APP/PS1 mice reveal that astrocytes do not migrate to amyloid-β plaques. Proc Natl Acad Sci U S A.

[79] Laping NJ, Teter B, Nichols NR, Rozovsky I, Finch CE (1994). Glial fibrillary acidic protein: regulation by hormones, cytokines, and growth factors. Brain Pathol.

[80] Tani M, Glabinski AR, Tuohy VK, Stoler MH, Estes ML, Ransohoff RM (1996). In situ hybridization analysis of glial fibrillary acidic protein mRNA reveals evidence of biphasic astrocyte activation during acute experimental autoimmune encephalomyelitis. Am J Pathol.

[81] Porchet R, Probst A, Bouras C, Dráberová E, Dráber P, Riederer BM (2003). Analysis of gial acidic fibrillary protein in the human entorhinal cortex during aging and in Alzheimer’s disease. Proteomics..

[82] Kraft AW, Hu X, Yoon H, Yan P, Xiao Q, Wang Y (2013). Attenuating astrocyte activation accelerates plaque pathogenesis in APP/PS1 mice. FASEB J.

[83] Alberdi E, Wyssenbach A, Alberdi M, Sánchez-Gómez MV, Cavaliere F, Rodríguez JJ (2013). Ca2+−dependent endoplasmic reticulum stress correlates with astrogliosis in oligomeric amyloid β-treated astrocytes and in a model of Alzheimer’s disease. Aging Cell.

[84] Thangavel R, Kempuraj D, Stolmeier D, Anantharam P, Khan M, Zaheer A (2013). Glia maturation factor expression in entorhinal cortex of Alzheimer’s disease brain. Neurochem Res.

[85] Yang C, Huang X, Huang X, Mai H, Li J, Jiang T (2016). Aquaporin-4 and Alzheimer’s disease. J Alzheimers Dis.

[86] Mestre H, Hablitz LM, Xavier AL, Feng W, Zou W, Pu T (2018). Aquaporin-4-dependent glymphatic solute transport in the rodent brain. Elife..

[87] Louveau A, Plog BA, Antila S, Alitalo K, Nedergaard M, Kipnis J (2017). Understanding the functions and relationships of the glymphatic system and meningeal lymphatics. J Clin Invest.

[88] Lan YL, Zhao J, Ma T, Li S (2016). The potential roles of aquaporin 4 in Alzheimer’s disease. Mol Neurobiol.

[89] Moftakhar P, Lynch MD, Pomakian JL, Vinters HV (2010). Aquaporin expression in the brains of patients with or without cerebral amyloid angiopathy. J Neuropathol Exp Neurol.

[90] Hoshi A, Yamamoto T, Shimizu K, Ugawa Y, Nishizawa M, Takahashi H (2012). Characteristics of aquaporin expression surrounding senile plaques and cerebral amyloid angiopathy in Alzheimer disease. J Neuropathol Exp Neurol.

[91] Pérez E, Barrachina M, Rodriguez A, Torrejón-Escribano B, Boada M, Hernández I (2007). Aquaporin expression in the cerebral cortex is increased at early stages of Alzheimer disease. Brain Res.

[92] Serrano-Pozo A, Gómez-Isla T, Growdon JH, Frosch MP, Hyman BT (2013). A phenotypic change but not proliferation underlies glial responses in Alzheimer disease. Am J Pathol.

[93] Pelvig DP, Pakkenberg H, Regeur L, Oster S, Pakkenberg B (2003). Neocortical glial cell numbers in Alzheimer’s disease. A stereological study. Dement Geriatr Cogn Disord.

[94] Zamanian JL, Xu L, Foo LC, Nouri N, Zhou L, Giffard RG (2012). Genomic analysis of reactive astrogliosis. J Neurosci.

[95] Liddelow Shane A., Guttenplan Kevin A., Clarke Laura E., Bennett Frederick C., Bohlen Christopher J., Schirmer Lucas, Bennett Mariko L., Münch Alexandra E., Chung Won-Suk, Peterson Todd C., Wilton Daniel K., Frouin Arnaud, Napier Brooke A., Panicker Nikhil, Kumar Manoj, Buckwalter Marion S., Rowitch David H., Dawson Valina L., Dawson Ted M., Stevens Beth, Barres Ben A. (2017). Neurotoxic reactive astrocytes are induced by activated microglia. Nature.

[96] Vainchtein Ilia D., Chin Gregory, Cho Frances S., Kelley Kevin W., Miller John G., Chien Elliott C., Liddelow Shane A., Nguyen Phi T., Nakao-Inoue Hiromi, Dorman Leah C., Akil Omar, Joshita Satoru, Barres Ben A., Paz Jeanne T., Molofsky Ari B., Molofsky Anna V. (2018). Astrocyte-derived interleukin-33 promotes microglial synapse engulfment and neural circuit development. Science.

[97] Bouvier DS, Murai KK (2015). Synergistic actions of microglia and astrocytes in the progression of Alzheimer’s disease. J Alzheimers Dis.

[98] Kaur D, Sharma V, Deshmukh R (2019). Activation of microglia and astrocytes: a roadway to neuroinflammation and Alzheimer’s disease. Inflammopharmacology..

[99] Yates SL, Burgess LH, Kocsis-Angle J, Antal JM, Dority MD, Embury PB (2000). Amyloid beta and amylin fibrils induce increases in proinflammatory cytokine and chemokine production by THP-1 cells and murine microglia. J Neurochem.

[100] Calsolaro Valeria, Edison Paul (2016). Neuroinflammation in Alzheimer's disease: Current evidence and future directions. Alzheimer's & Dementia.

[101] Gomez-Arboledas A, Davila JC, Sanchez-Mejias E, Navarro V, Nuñez-Diaz C, Sanchez-Varo R (2017). Phagocytic clearance of presynaptic dystrophies by reactive astrocytes in Alzheimer’s disease. Glia.

